# Meningococcal Pneumonia in a Young Healthy Male

**DOI:** 10.1155/2018/2179097

**Published:** 2018-08-27

**Authors:** Abdullah M. Al Alawi

**Affiliations:** ^1^Division of Medicine, Royal Darwin Hospital, Darwin, NT, Australia; ^2^Department of Medicine, Sultan Qaboos University Hospital, Muscat, Oman

## Abstract

A 23-year-old male presented to the emergency department with one-day history of right-sided pleuritic chest pain, haemoptysis, and fever. In the emergency department, the blood pressure was 140/60 mmHg, heart rate 89/min, body temperature 40°C, respiratory rates 20 breaths/min, and oxygen saturation 98% in room air. Physical examination revealed rales and bronchial breathing in the right infrascapular region. Laboratory analysis showed raised white blood cell counts and elevated inflammation markers. Chest X-ray showed right lower lobe consolidation. Intravenous(IV) ceftriaxone and doxycycline were started for the management of community-acquired pneumonia as per the local guideline. Later, on admission, blood culture was positive for *Neisseria meningitidis* (*N. meningitidis*). Ceftriaxone was continued for 4 days, and the patient was discharged while being on oral amoxicillin (1 gm TDS) for another 3 days. He remained well during the outpatient follow-up.

## 1. Background

Meningococcal disease is a rare disease, and it affects mostly infants and young adults [1].The most common manifestation of meningococcal disease is meningitis (in 50% of cases), followed by bacteraemia (40% of cases), and fulminant disease (10–20% of cases) which is known to be associated with high mortality (about 50%) [2, 3]. Meningococcal pneumonia is an uncommon disease. The first cases were described by Jacobitz in 1907 when he demonstrated the presence of *N. meningitidis* in sputum samples obtained from 12 patients with pneumonia [4]. During the 1918 influenza pandemic, large outbreaks of meningococcal pneumonia were reported. Recently, Vossen et al. identified a total of 344 reported cases of meningococcal pneumonia between 1906 and 2015 [5]. Serogroup Y was found to be responsible for majority of meningococcal pneumonia followed by serogroup W [5, 6]. Recent influenza infection or preceding pharyngitis has been linked to meningococcal pneumonia [7, 8]. Old patients and patients with underlying diseases such as malignancy, cirrhosis, and diabetes mellitus are at an increased risk of contracting meningococcal pneumonia [9]. Other risk factors include smoking, asplenism, specific genetic predisposition, certain immunodeficiency disorders, and chronic lung diseases such as chronic obstructive pulmonary disease and bronchiectasis [2, 5]. The clinical presentation of meningococcal pneumonia is similar to the presentation of community-acquired pneumonia caused by other pathogens. Symptoms and signs include fever (50%), cough (31%), chills (50%), pleuritic chest pain, and dyspnea (23%) [5, 9, 10].

## 2. Case Presentation

A 23-year-old male presented to the emergency department with one-day history of right-sided pleuritic chest pain, haemoptysis, and fever. He had no history of a recent travel or contact with sick individuals. The patient had no significant medical background, and he was not taking any regular medication.

On admission, blood pressure was 140/60 mmHg, heart rate 89/min, body temperature 40°C, respiratory rates 20 breaths/min, and oxygen saturation 98% in room air. Physical examination revealed rales and bronchial breathing in the right infrascapular region. There was no clinical evidence of meningitis.

### 2.1. Investigations

Laboratory analysis showed the following results: haemoglobin level 146 g/L (normal 140–175), platelets count 373 × 10^9^/L (normal),white blood cell counts 19.6 × 10^9^/L (normal 3.5–10.0) (90% neutrophils and 10% lymphocytes), sodium 140 mmol/L (normal 135–145), potassium 3.6 mmol/L (normal 3.5–4.5), urea 3.7 mmol/L (normal 2.5–7.0), creatinine 104 µmol/L (normal 50–100), eGFR 87 ml/min/1.7 m^2^ (normal > 90), C-reactive protein at 58.5 mg/L (normal < 3), and an unremarkable liver function test. Chest X-ray demonstrated right lower lobe consolidation. With the history of haemoptysis and pleuritic chest pain, computed tomography pulmonary angiogram (CTPA) was performed, and it did not show pulmonary embolism (PE).

Sputum culture was found to be positive for oropharyngeal *Candida* species. However, a day later, *N*. *meningitidis* grew in one blood culture bottle, and it was sensitive to penicillin and ceftriaxone. Using polymerase chain reaction (PCR), we have identified *N*. *meningitidis* serogroup Y. Subsequently, two repeat sets of blood cultures, after initiation of antibiotics, were sent and reported negative. Additional results included undetectable urinary *Streptococcus* and *Legionella pneumophila* serogroup 1 antigens and a negative HIV serology test.

### 2.2. Treatment

The patient was started on 2 g of IV ceftriaxone and 100 mg of doxycycline as per the hospital guidelines for the management of community-acquired pneumonia. However, doxycycline was discontinued after day 1 after the blood culture result. In total, the patient received 4 days of IV ceftriaxone followed by 3 days of oral amoxicillin (1 gm,TDS). The Centers for Disease Control and Prevention was contacted to arrange chemoprophylaxis for the patient's contacts.

### 2.3. Outcome and Follow-Up

On day 4, the patient was discharged from the hospital and was reviewed at an outpatient clinic two weeks later. He showed a complete resolution of his symptoms.

## 3. Discussion

Diagnosis of meningococcal pneumonia might be challenging due to similarities of the symptoms with other bacterial pneumonia. Also, culture-based detection of *N. meningitidis* in the sputum sample could be misleading because *N. meningitidis* may be isolated as a part of the upper respiratory tract flora of the asymptomatic carrier [11]. However, there are several diagnostic strategies and guidelines developed to distinguish between the presence of *N. meningitidis* as a part of the normal flora versus pathogenic *N. meningitidis*. These guidelines take into account several factors including the quantity of *N. meningitidis* compared to other bacteria and the presence of inflammatory cells in the sputum sample [5, 12]. In addition, blood culture is an insensitive test to diagnose meningococcal pneumonia because of low incidence of bacteraemia in patients with meningococcal pneumonia compared to patients with pneumococcal pneumonia [10]. Using sensitive and rapid techniques such as polymerase chain reaction (PCR) to detect *N. meningitidis* DNA in blood offers an advantage over the culture, but this test has not been fully validated for a routine clinical use [13, 14]. Elevated white cell counts and inflammation markers such as C-reactive protein are commonly seen in patients with meningococcal pneumonia [5]. Chest X-ray abnormalities may include unilateral infiltration (70% of cases), bilateral infiltrations (20%), and pleural effusion (10%) [6].

Early antibiotic treatment is the most effective therapy in meningococcal disease. Prior to 1990, majority of cases were treated with penicillin. However, after 1990, cephalosporins were used due to the emergence of penicillin-resistant strains and high lethality of untreated meningococcal disease (16%) [5]. However, penicillin resistance among *N. meningitidis* isolates is uncommon [15, 16]. In the previously reported cases, the duration of treatment varied between 5 days and 8 weeks (in a patient with complication of pericarditis) [6].

Complications of meningococcal pneumonia are uncommon and include septic shock, lung abscess, pleural effusion, and pericarditis [6]. Factors associated with poor prognosis include old age, presence of comorbid conditions, and pneumonia caused by serogroup W [5, 17].

## 4. Conclusion

Meningococcal pneumonia is a rare manifestation of *N. meningitidis*, and the diagnosis can be challenging because of low sensitivity of the blood culture and lack of specificity of the sputum sample because of the carrier status of asymptomatic people. Early initiation of antibiotics is very crucial due to the high mortality rate of untreated meningococcal disease especially in elderly patients and patients with underlying comorbidities. Further research studies are needed to develop diagnostic tests with better sensitivity and specificity. In addition, developing vaccinations that cover different serogroups would be valuable in reducing the global burden of the disease.
